# Use of a Chagas Urine Nanoparticle Test (Chunap) to Correlate with Parasitemia Levels in *T*. *cruzi*/HIV Co-infected Patients

**DOI:** 10.1371/journal.pntd.0004407

**Published:** 2016-02-26

**Authors:** Yagahira E. Castro-Sesquen, Robert H. Gilman, Carolina Mejia, Daniel E. Clark, Jeong Choi, Melissa J. Reimer-McAtee, Rosario Castro, Edward Valencia-Ayala, Jorge Flores, Natalie Bowman, Ricardo Castillo-Neyra, Faustino Torrico, Lance Liotta, Caryn Bern, Alessandra Luchini, The Chagas/HIV Working Group in Bolivia and Peru

**Affiliations:** 1 Laboratorio de Investigación en Enfermedades Infecciosas, Universidad Peruana Cayetano Heredia, Lima, Peru; 2 Center for Applied Proteomics and Molecular Medicine, George Mason University, Manassas, Virginia, United States of America; 3 Department of International Health, Bloomberg School of Hygiene and Public Health, Johns Hopkins University, Baltimore, Maryland, United States of America; 4 Colectivo de Estudios Aplicados, Desarrollo Social, Salud y Medio Ambiente, Cochabamba, Bolivia; 5 Department of Internal Medicine and Pediatrics, Vanderbilt University, Nashville, Tennessee, United States of America; 6 Division of Cardiovascular Medicine, University of California Davis, Davis, California, United States of America; 7 Tulane University Medical Center, New Orleans, Louisiana, United States of America; 8 Servicio de Infectología, Hospital Clínico Viedma, Cochabamba, Bolivia; 9 Hospital San Juan De Dios, Santa Cruz de la Sierra, Bolivia; 10 School of Medicine, Department of Medicine, University of North Carolina, Chapel Hill, Chapel Hill, North Carolina, United States of America; 11 Perelman School of Medicine, University of Pennsylvania, Philadelphia, Pennsylvania, United States of America; 12 Facultad de Medicina, Universidad Mayor de San Simón, Cochabamba, Bolivia; 13 Global Health Sciences, Department of Epidemiology and Biostatistics, School of Medicine, University of California, San Francisco, San Francisco, California, United States of America; US Food and Drug Administration, UNITED STATES

## Abstract

**Background:**

Early diagnosis of reactivated Chagas disease in HIV patients could be lifesaving. In Latin America, the diagnosis is made by microscopical detection of the *T*. *cruzi* parasite in the blood; a diagnostic test that lacks sensitivity. This study evaluates if levels of *T*. *cruzi* antigens in urine, determined by Chunap (Chagas urine nanoparticle test), are correlated with parasitemia levels in *T*. *cruzi*/HIV co-infected patients.

**Methodology/Principal Findings:**

*T*. *cruzi* antigens in urine of HIV patients (N = 55: 31 *T*. *cruzi* infected and 24 *T*. *cruzi* serology negative) were concentrated using hydrogel particles and quantified by Western Blot and a calibration curve. Reactivation of Chagas disease was defined by the observation of parasites in blood by microscopy. Parasitemia levels in patients with serology positive for Chagas disease were classified as follows: High parasitemia or reactivation of Chagas disease (detectable parasitemia by microscopy), moderate parasitemia (undetectable by microscopy but detectable by qPCR), and negative parasitemia (undetectable by microscopy and qPCR). The percentage of positive results detected by Chunap was: 100% (7/7) in cases of reactivation, 91.7% (11/12) in cases of moderate parasitemia, and 41.7% (5/12) in cases of negative parasitemia. Chunap specificity was found to be 91.7%. Linear regression analysis demonstrated a direct relationship between parasitemia levels and urine *T*. *cruzi* antigen concentrations (p<0.001). A cut-off of > 105 pg was chosen to determine patients with reactivation of Chagas disease (7/7). Antigenuria levels were 36.08 times (95% CI: 7.28 to 64.88) higher in patients with CD4+ lymphocyte counts below 200/mL (p = 0.016). No significant differences were found in HIV loads and CD8+ lymphocyte counts.

**Conclusion:**

Chunap shows potential for early detection of Chagas reactivation. With appropriate adaptation, this diagnostic test can be used to monitor Chagas disease status in *T*. *cruzi*/HIV co-infected patients.

## Author Summary

Reactivation of Chagas disease in people living with HIV is a serious clinical condition that is associated with high mortality. Hence, early diagnosis and treatment can be lifesaving. Although there are not well accepted criteria to identify patients at risk of reactivation, parasitemia levels are usually considered as the best predictor. Microscopy is used in Latin America for detection of parasitemia levels. However, this has low sensitivity, which usually leads to a delay in diagnosis and treatment. Quantitative PCR is used only for research proposes in endemic areas. Antigens in urine (antigenuria) are correlated with parasitemia levels in animal models, as well as in cases of congenital Chagas disease. We believe that antigenuria can also be used for prediction of parasitemia levels in *T*. *cruzi*/HIV co-infected patients. In this study, Chunap (Chagas urine nanoparticle test) was used for concentration and quantification of *T*. *cruzi* antigens in urine of *T*. *cruzi*/HIV co-infected patients. Values of more than 105 pg of *T*. *cruzi* antigens in urine were observed only in patients with reactivation of Chagas disease. This study shows that antigenuria levels are highly correlated to levels of parasitemia and can be used as a non-invasive technique for monitoring parasitemia levels in *T*. *cruzi*/HIV co-infected patients.

## Introduction

Chagas disease, caused by the protozoan *Trypanosoma cruzi*, affects an estimated 7.8 million people in the Americas [1]. Similar to HIV infection, Chagas disease is most prevalent in the adult population [2]. Massive rural-to-urban migration throughout has brought many cases of chronic Chagas disease into the city where patients are at risk for acquisition of HIV. This has created conditions for emergence of *T*. *cruzi*/HIV co-infection as a significant public health problem in the Americas.

Bolivia has the highest prevalence of *T*. *cruzi* infection in the world; with adult seroprevalence figures of up to 30% in urban areas and up to 80–90% in some rural areas [3, 4]. HIV infection remains under-diagnosed in Bolivia and there are no data about the epidemiology of *T*. *cruzi*/HIV co-infection in this country.

After infection with *T*. *cruzi*, immunocompetent patients enter the acute phase, this phase is characterized by high parasitemia, mild and nonspecific febrile illness, and, rarely, life-threatening myocarditis and/or meningoencephalitis [5]. After 2 to 3 months, patients pass into the chronic phase, which is characterized by positive serology but microscopically undetectable parasitemia. The chronic phase persists life-long in the absence of successful treatment. Many people in the chronic phase will remain asymptomatic throughout life, but 20% will develop cardiomyopathy or mega-syndromes of the digestive tract [6]. Chagas disease is usually acquired during childhood in endemic regions, in contrast to HIV infection [7]. However, the chronic manifestations are not seen until adulthood.

An estimated 20% of *T*. *cruzi/*HIV co-infected individuals develop *T*. *cruzi* reactivation. Presentation includes high levels of parasitemia and severe clinical manifestations; usually involving CNS syndromes (50–85%) and/or myocarditis (10–55%) [7–12]. Alterations in the CNS include meningoencephalitis and/or brain accesses that appear very similar, by neuroimaging, to those produced by *Toxoplasma gondii* reactivation. As such, direct detection of the parasite is needed to confirm the diagnosis. Mortality in patients with meningoencephalitis reaches 80–100%, partly as a consequence of late diagnosis and treatment [7].

Some studies suggest that early diagnosis and treatment with both benznidazole and combination antiretroviral therapy (cART) could be lifesaving in patients with CNS reactivation [7, 13–14]. However, there are no well accepted criteria to identify patients at risk of reactivation. Serology is the standard diagnostic modality in the chronic phase, but does not distinguish between *T*. *cruzi* infection with and without reactivation. Current criteria for reactivation are based on microscopic observation of the parasite in blood, but because of its low sensitivity, this technique detects reactivation when the parasitemia is high [15]. By this time, symptoms may be severe and rescue treatment is likely to fail [15, 16]. Furthermore, microscopy requires extensive training in specimen preparation, and discordant readings by microscopists are frequent. Blood culture and xenodiagnosis have higher sensitivity but take 20–60 days to give conclusive results; both are rarely used for diagnosis [15]. Quantitative polymerase chain reaction (qPCR) has been suggested as a highly efficient method for monitoring levels of parasitemia in *T*. *cruzi*/HIV co-infected patients [15].

The qPCR is used to monitor levels of parasitemia as well as risk of reactivation in immunocompromised individuals after organ transplantation in the USA [17,18]. However, in Latin America, qPCR is not routinely used. A study with *T*. *cruzi*/HIV co-infected patients from Brazil demonstrated that the majority of *T*. *cruzi*/HIV co-infected patients have substantially higher parasitemia levels when compared to immunocompetent *T*. *cruzi*-infected individuals. Some of these patients had detectable parasitemia by microscopy even in the absence of symptoms [15]. These asymptomatic patients do not appear to have greater short-term mortality, but they are hypothesized to be at increased risk of developing symptomatic reactivation [15].

High levels of parasitemia may represent an intermediate phase that precedes clinical reactivation. In such circumstances, preemptive treatment may be justified [10, 15]. However, systematic data on this hypothesis is lacking. Detection of urine *T*. *cruzi* antigens has been shown to correlate with parasitemia levels in animals [19] and could be a convenient, non-invasive tool to monitor levels of parasitemia in HIV patients. However, antigens in urine exist at very low concentrations; below the limit of detection of conventional immunoassays. Furthermore, antigens are masked by highly abundant resident proteins, and are rapidly degraded by endogenous and exogenous enzymes [20–25].

A novel nanotechnology based on the use of nano-porous particles that contain high affinity chemical baits (trypan blue) in the inner core is proposed for concentration and preservation of antigens in urine [20–25]. This technology (Chagas urine nanoparticle test, Chunap) has been applied in the direct diagnosis of congenital Chagas disease with excellent agreement with standard diagnostic tests [26]. Nano-porous particles are synthetized with poly (N-isopropyl acrylamide) (pNIPAm) and N,N′-methylenebisacrylamide (BAAm) and coupled with chemical baits via amidation reaction. The nano-porous structure of the particles performs size sieving, allowing proteins to penetrate inside the particles, depending on their molecular weight and their dimensional shape. The trypan blue inside the particles captures proteins with extremely high affinity (K_D_ < 10^−12^ M) within minutes [20–26].

A sensitive but relatively simple noninvasive test for monitoring *T*. *cruzi* infection in HIV co-infected is needed. This tool could lead to early treatment and may be lifesaving. In this study, we demonstrate that levels of *T*. *cruzi* antigens determined by Chunap are associated with levels of parasitemia in *T*. *cruzi*/HIV co-infected patients and could be a valuable non-invasive tool for monitoring Chagas disease reactivation in HIV co-infected patients.

## Methods

### Ethics statement

The protocols were approved by the institutional review boards of the study hospitals (Hospital Clínico Viedma, Centro de Vigilancia y Referencia de HIV/AIDS, the Instituto de desarrollo Humano and the Colectivo de Estudios Aplicados, Desarrollo Social, Salud y Medio ambiente in Cochabamba, and the Universidad Catolica in Santa Cruz, Bolivia), Asociacion Benefica Prisma (Lima, Peru) and the Johns Hopkins University (Baltimore, MD).

### Human study design

We evaluated 55 samples of HIV patients (31 *T*. *cruzi*-infected and 24 *T*. *cruzi* uninfected) from Cochabamba and Santa Cruz, Bolivia. A written informed consent was obtained from all participants. The diagnosis of HIV infection was performed according to the Bolivian National Control Program of HIV/AIDS, and was based on detection of specific antibodies by an ELISA test (Vironostika HIV UNIformII Ag/Ab, Biomerieux) and Western blot (New Lab Blot I, BioRad). Blood and urine samples were obtained early on in hospitalization and none had received *T*. *cruzi* treatment. Confirmation of *T*. *cruzi* infection was based on positive results by 2 or more of the following commercial tests: Chagatest ELISA (Wienner Lab, Rosario- Argentina, sensitivity: 98.81% and specificity: 99.62%), Chagatest ELISA recombinant v 3.0 (Wienner Lab, Rosario, Argentina; sensitivity: 99.3% and specificity: 98.7%), and the indirect hemagglutination test (IHA) (PolyChaco. Sensitivity: 98%, Specificity: 99%).

### Determination of HIV load and immunosuppression status

Medical records of each participant were reviewed to obtain data on HIV load, CD4+ and CD8+ T-cell counts. Most patients were recently diagnosed with HIV and were not receiving ART at the time of diagnosis. As a result, high viral loads were observed in patients with and without Chagas disease (mean: 220969.5 and 84960.6 copy number/ml blood, respectively). We therefore used a classification of high HIV load as follows: 1) <5000 copies/ml, 2) ≥5000–30 000 copies/ml, and 3) >30 000 copies/ml [27, 28]. The immunosuppression status was determined by the levels of CD4+ T-cell counts according to CDC classification system as follow: 1) ≤ 200, 2) 201–500 and 3) ≥ 500 [29].

### Chagas disease status

Reactivation of *T*. *cruzi* infection was determined by detection of circulating parasites by micromethod. In this technique, blood samples are collected in 4–6 heparinized microhematocrit tubes, centrifuged and the buffy coat layer is examined microscopically for parasites [30]. Quantification of the number of copies of DNA of the parasite in blood was performed by quantitative PCR [31–34]. Patients with Chagas disease were considered to be in one of three categories according to the levels of parasitemia: High parasitemia or cases with reactivation of Chagas disease (n = 7, patients with positive micromethod and PCR), moderate parasitemia (n = 13, patients with positive PCR but negative micromethod) and negative parasitemia (n = 12, patients with negative PCR but positive by serology).

### Pre-analytical handling of urine samples and urinalysis

Patients were asked to provide the first urine of the day before ingestion of liquids, where midstream specimens were collected. Urinalysis was done using urine test strips (Multistik 10 SG, Siemens, NY-USA). Mean values of urine specific gravity was within normal levels (mean: 1.022, SD: 0.008). Urine samples (10 mL) were immediately centrifuged after collection at 3000 rcf for 10 min and the supernatant was stored in liquid nitrogen or -80°C until use. For antigen detection the supernatant was adjusted to pH 5–6 with 1M HCl.

### Concentration of *T*. *cruzi* antigens using hydrogel nano-porous particles

Poly N-isopoprylacrylamide (NIPAm) particles coupled with trypan blue dye (Poly (NIPAm/TB)) were synthesized as previously described [20–26]. Urine samples (10 mL) were incubated with 1 mL of poly (NIPAm/TB) particle suspension (7.2 mg/ml dry weight) for 30 min at room temperature under rotation. Capturing, concentration, and elution of antigens from the particles was done as previously described [26]. Eluates were mixed with 10 μl of 250 mg/mL trehalose (Fluka Chemicals, MO-USA) solution and 10 μl of 1% (v/v) red food dye (McCormick, MD-USA) in MilliQ water and dried under nitrogen flow (Organomation). Dried eluates were suspended in 40 μl of SDS sample buffer (50 mM TrisHCl pH 6.8, 2% SDS, 1% 2-mercaptoethanol, 10% glycerol and 0.02% bromophenol blue). The effective concentration factor is 250 fold based on volumetric ratio (initial volume / final volume = 10000 μl /40 μl).

### Determination of levels of *T*. *cruzi* antigens by Western blot

Aliquots of 20 μl of re-suspended antigens were heated to 100°C for 7 min. Electrophoresis and Western Blot analysis of the antigens were performed as previously described [26]. Briefly, antigens were detected with an anti-*T*. *cruzi* lipophosphoglycan (LPG) mouse monoclonal antibody (Cedarlane Laboratories USA Inc, NC-USA), diluted 1:250 in PBS with 0.2% I-Block and 0.1% Tween 20. After six washing steps with PBS supplemented with 0.1% Tween 20, antigens were incubated with peroxidase conjugated goat anti-mouse IgM (Invitrogen Corporation, CA-USA) diluted 1:5000 in PBS supplemented with 0.2% I-Block and 0.1% Tween 20, for 60 minutes at room temperature. The molecular weight was determined using MagicMark XP Western Protein Standard (Invitrogen Corporation, CA-USA). Each sample was run twice. Visualization of antigenic bands was done using an enhanced chemiluminescence system (Supersignal West Dura, Thermo Fisher Scientific, MA-USA). The trypomastigote excretory-secretory antigen (TESA) was used to develop a calibration curve. This antigen was harvested from cell cultures of *T*. *cruzi* Y strain in LLC-MK2 cells, as previously described [31]. The calibration curve was established using the following TESA antigen concentrations: 5 pg, 10 pg, 50 pg, 250 pg and 500 pg (R^2^ = 0.95) (S1 Fig). Antigen levels were determined by densitometry of western blots using myImageAnalysis Software (Thermo Scientific, USA) of five specific bands (22 kDa, 42 kDa, 58 kDa, 75 kDa and 82 kDa) that were detected by Western blot. The limit of detection of the test in normal urine samples spiked with *T*. *cruzi* antigens was 10 pg/ml. The presence of any of the five diagnostic bands (22 kDa, 42 kDa, 58 kDa, 75 kDa and 82 kDa) was considered as a positive result. In each experiment we included a negative control (urine sample of healthy volunteer) and a positive control (10 ml of healthy volunteer urine sample containing 1 ng of TESA antigen). The Chunap was carried out by a laboratory biologist who was blinded to the Chagas status of the patient.

### DNA extraction and qPCR

qPCR was performed to evaluate levels of parasitemia. DNA was purified from 500 μl of blood clot samples as previously described [32, 33]. The quantification of DNA was determined by spectrophotometry using a Nanodrop 2000 instrument (Thermo Scientific, Delaware, USA) and only samples with a ratio of 260nm/280nm of ~1.8 were used for PCR analysis. qPCR was performed using published methods [34] with the modifications detailed before [35]. The qPCR was carried out by a laboratory biologist who was blinded to the Chagas status of the patient.

### Statistical analyses

STATA 13 software was used for all statistical analysis. Parasitemia levels determined by qPCR were evaluated in a logarithmic scale. Differences in mean levels of parasitemia, CD4+ and CD8+ T cell counts, and HIV load between patients with and without reactivation were evaluated by Student’s t-test with equal and unequal variances. Receiver Operating Characteristic (ROC) analysis was used to determine the sensitivity and specificity of different cut-offs of antigenuria and parasitemia levels for the diagnosis of reactivation of Chagas disease using microscopy as gold standard. The association between urine *T*. *cruzi* antigen concentration and parasitemia levels was evaluated using a linear regression model unadjusted and adjusted by sex, age (years), antiretroviral treatment (yes versus no), HIV load and immune status (CD4+ and CD8+ T cell counts). The use of a sample size of 31 *T*. *cruzi*/HIV co-infected patients with a significance level of 0.05 gives a statistical power of 72% to determine associations between levels of antigenuria and parasitemia. This sample size gives a power below 70% to determine other statistical associations.

## Results

### Characteristics of patients

The nested-case control study consisted of 55 HIV patients (31 *T*. *cruzi* infected and 24 *T*. *cruzi* non-infected). Mean age was 36.8 years (SD: 15.1 years). Mean levels of HIV loads were considerably higher (187387.5 copies/ml, SD: 549436.2 copies/ml) [27, 28], which could be explained by the short duration of HIV diagnosis (mean: 28.3 months, SD: 24.9 months). Mean levels of CD4+ cell and CD8+ cell counts were within normal limits according to the CDC classification (301.3 cells, SD: 226.2 cells, and 765.9 cells, SD: 543.4 cells, respectively) [29]. Tuberculosis was the most frequent co-infection in these patients (n = 6 cases). Characteristics of patients stratified by Chagas status are shown in Table 1. There were no significant differences in sex, HIV load, CD4+ cell count, CD8+ cell count, weight, time of HIV diagnosis, and presence of co-infections. There was however a strong trend for HIV patients co-infected with Chagas to be older (p = 0.06).

**Table 1:** Characteristics of HIV patients in the nested-case control study.

	Positive Serology for Chagas Disease	Negative Serology for Chagas disease	p-value
Sex (n, percentage)			0.92
Female	15 (27.27%)	12 (21.82%)	
Male	16 (29.09%)	12 (21.82%)	
Age in years (mean, SE)	40.6 (3.6)	31.9 (2.2)	0.06
HIV load in copies/ml (mean, SE)	220969.5 (136363.3)	84960.6 (50325.4)	0.36
Count CD4+ cells (mean, SE)	273.0 (47.4)	337.4 (53.3)	0.37
Count CD8+ cells (mean, SE)	782.1 (121.7)	745.5 (111.0)	0.83
Weight in kg (mean, SE)	59.7 (1.8)	60.3 (2.9)	0.85
Time from HIV diagnosis months (mean, SE)	27.34 (5.1)	29.42 (5.8)	0.79
Co-infections (n, %)^a^			
Total	7 (16.27%)	3 (6.97%)	0.30
Active tuberculosis	4	2	
Enteroparasitosis	2	2	
Pneumonia	1	0	
Malaria	1	0	
Herpes Zoster	1	0	
Histoplasmosis	0	1	

^a^ Some patients showed more than one co-infection during the time of evaluation.

### Performance of Chunap in the diagnosis of Chagas disease

**Fig 1:**
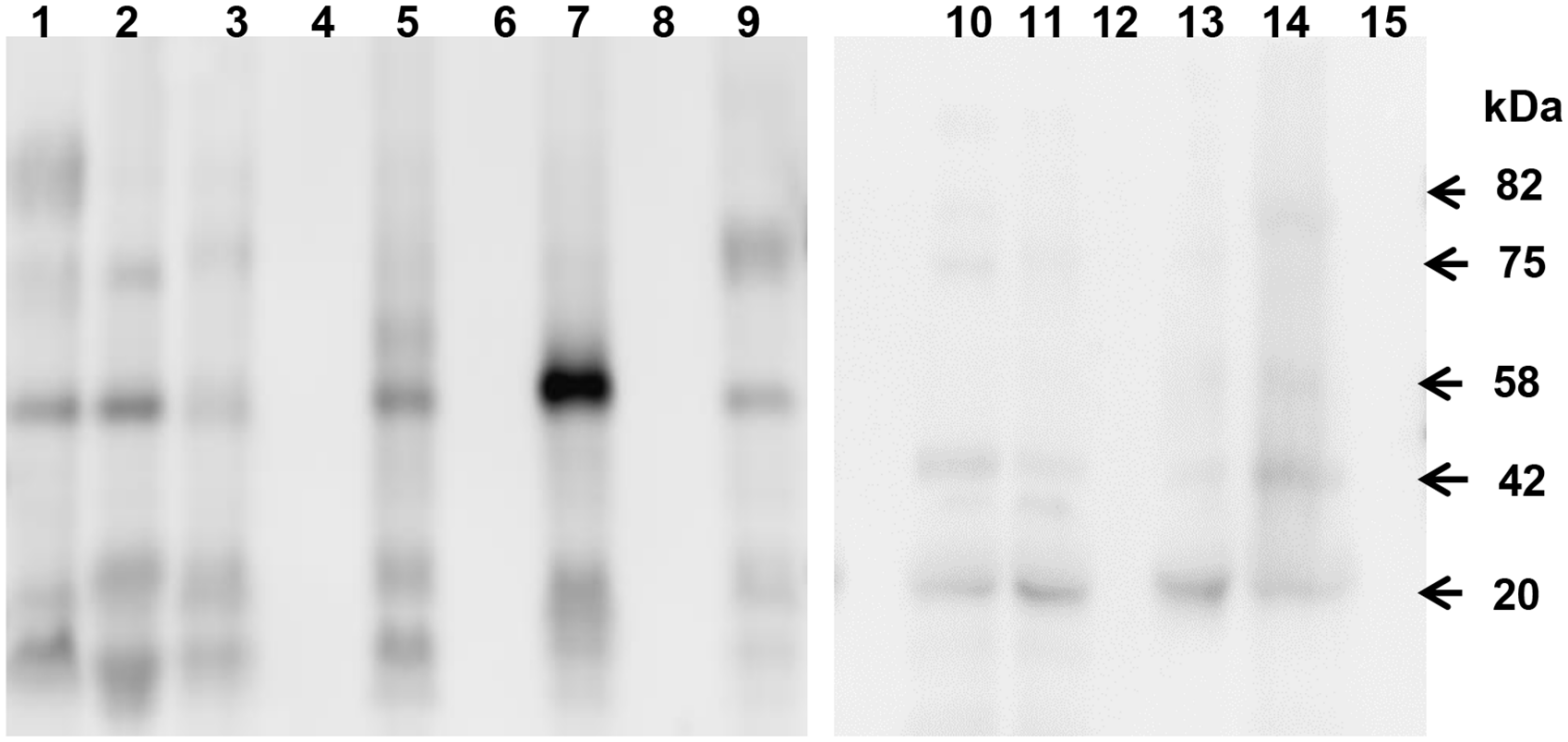
Antigenic bands in nanoparticles-concentrated urine samples of patients with HIV*/T*. *cruzi* co-infection. Bands were detected by Western Blot using a monoclonal antibody anti-lipophosphoglycan of *T*. *cruzi*. Bands of 20 kDa, 42 kDa, 58 kDa, 75 kDa, 82 kDa were considered specific for *T*. *cruzi*. Bands of other molecular weight were not considered for diagnosis criteria. Urine samples of *T*. *cruzi*+/HIV+ patients: Lanes 1–3, 5, 7, 9, 10–11, 13–14. Urine samples of *T*. *cruzi*-/HIV+ patients: Lanes 4, 6, 8, 12, and 15.

In this study poly (NIPAm)/TB nanoparticles were used to increase the effective sensitivity of western blot analysis in the detection of *T*. *cruzi* antigens by 100 fold as previously described [26]. Bands of 22 kDa, 42 kDa, 58 kDa, 75 kDa and 82 kDa were detected in nanoparticle-concentrated urine samples of *T*. *cruzi*/HIV co-infected patients (Fig 1). Bands of 22 kDa, 42 kDa and 55 kDa were also detected in 2 (2/24) urine samples of *T*. *cruzi*-uninfected/HIV+ patients yielding a specificity of 91.7%. One of these two Chagas negative patients also had a co-infection with *M*. *tuberculosis*. Other bands were also recognized in urine samples of *T*. *cruzi* infected patients, but the specificity was below 60% and were therefore excluded as potential diagnosis criteria. The percentage of positive results detected by Chunap was 100% (7/7) among the cases with reactivation or high parasitemia, 91.7% (11/12) among cases with moderate parasitemia, and 41.7% (5/12) among patients with negative parasitemia (Table 2). The percentage of *T*. *cruzi*-infected patients detected by Chunap, compared to those positive by microscopy, PCR, and ELISA, was 100% (7/7), 95% (18/19) and 74% (23/31), respectively. See S1 Diagram.

**Table 2:** Percentages of positive patients detected by Chunap stratified by levels of parasitemia among HIV*/T*. *cruzi* infected patients.

	Chunap	Micromethod	qPCR	Serology
Reactivation or high parasitemia (n = 7)	7 (100%)	7 (100%)	7 (100%)	7 (100%)
Moderate parasitemia (n = 12)	11 (91.7%)	0 (0%)	12 (100%)	12 (100%)
Negative parasitemia (n = 12)	5 (41.7%)	0 (0%)	0 (0%)	12 (100%)

Confirmation of *T*. *cruzi* infection was based on positive results by 2 or more of serological tests for detection of anti-*T*. *cruzi* IgG.

### Chunap and correlation with levels of parasitemia

**Fig 2:**
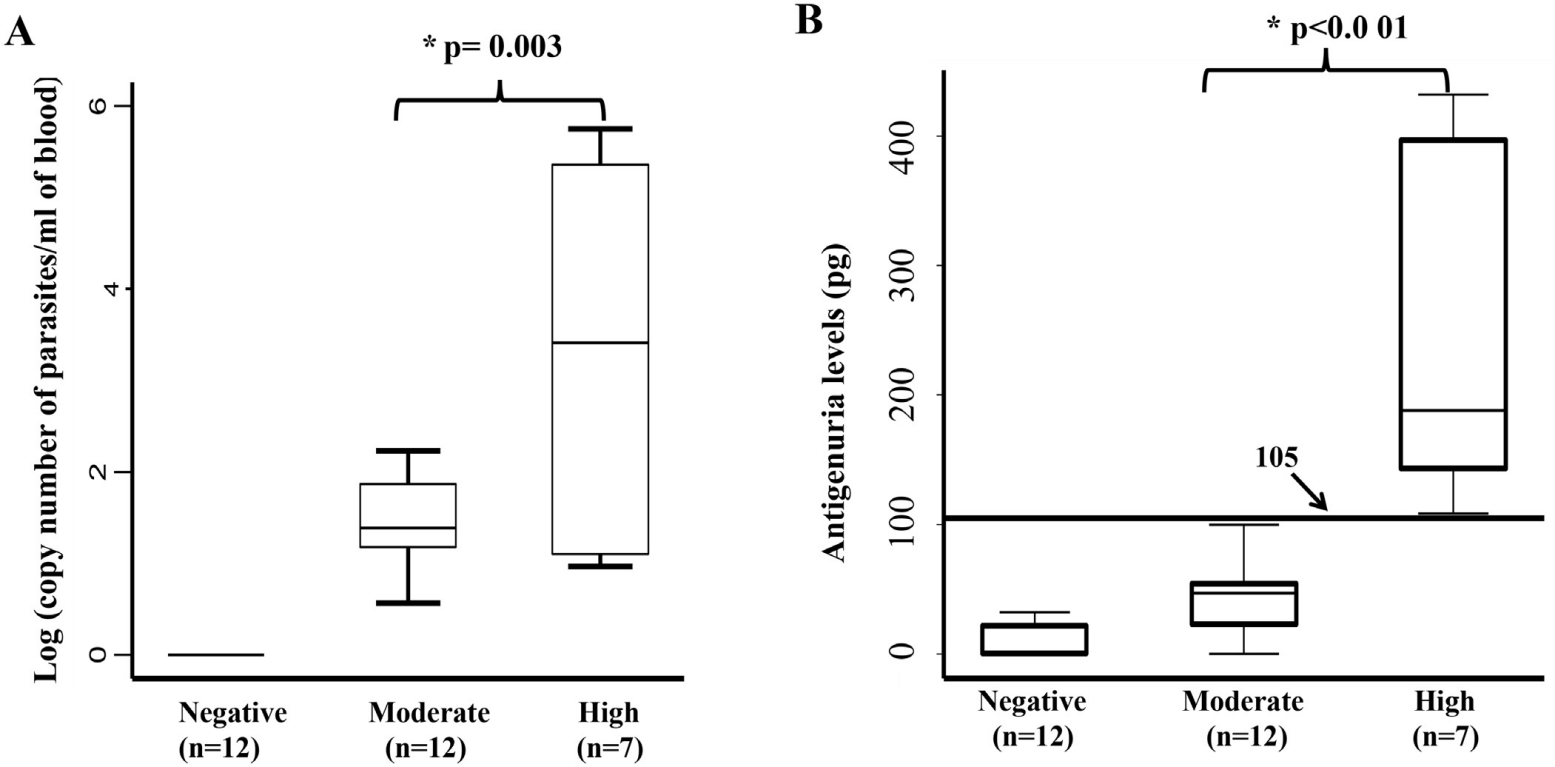
Levels of parasitemia and antigenuria in *T*. *cruzi*/HIV co-infected patients. A.) Levels of parasitemia determined by qPCR in *T*. *cruzi*/HIV co-infected patients. B.) Levels of antigenuria determined by Chunap in the three groups of *T*. *cruzi*/HIV co-infected patients. Levels of parasitemia: High parasitemia = positive by PCR and microscopy, Moderate parasitemia = positive by PCR, and negative by microscopy, Negative parasitemia = negative by PCR and microscopy but positive by serology, and No Chagas = negative by microscopy, PCR and serology. Bottom and top limits of the boxes correspond to first and third quartiles, and the line inside the boxes represents the second quartile (median). P-values were calculated using T-test with equal variances. * Statistically significant difference.

Mean levels of parasitemia were significantly different between patients with reactivation of Chagas disease (3.28 logarithm copy number of parasites/ml, 95% CI: 1.56 to 5.00) and patients without reactivation but with positive qPCR (1.43 logarithm copy number of parasites/ml, 95% CI: 1.13 to 1.72) (p = 0.003) (Table 3) (Fig 2A). Similarly, mean levels of antigenuria were significantly higher in patients with high parasitemia or reactivation of Chagas disease (mean = 242.21pg, 95% CI: 125.45 to 358.95) compared to patients with moderate parasitemia (mean = 43.32 pg, 95% CI: 25.06 to 61.58) (p<0.001) (Fig 2B). Using 105 pg as a cut-off, Chunap could detect all patients with reactivation (7/7). The best balance between specificity (90.62%, 3/29) and sensitivity (71.43%, 5/7) for determination of reactivation by qPCR was obtained with the cut-off of 2 log (parasites/ml blood), this cut-off was used for categorization of parasitemia levels in the regression model. High variability of parasitemia levels measured by qPCR was observed, even in a logarithmic scale.

**Table 3:** Linear regression model for prediction of levels of antigenuria by levels of parasitemia, and relationship with immune status, HIV load, sex, age and initiation of antiretroviral therapy.

	Unadjusted			Adjusted		
	Coefficient	95% CI	p-value	Coefficient	95% CI	p-value
**Log parasitemia**^a^						
< 2.0	31.52	17.04 to 46.01	<0.001	33.65	17.48 to 49.82	<0.001
≥ 2.0	106.00	85.13 to 126.85	<0.001	101.42	79.98 to 122.86	<0.001
**CD4 classification**^b^						
>500 cells	Reference			Reference		
200–500 cells	-27.63	-94.80 to 39.54	0.412	-0.30	7.28 to 64.88	0.983
< 200 cells	22.37	-45.56 to 90.28	0.511	36.08	7.28 to 64.88	0.016
**CD8 classification**^c^						
> 1000 cells	Reference			Reference		
500–1000 cells	-4.29	-69.39 to 60.81	0.895	7.71	-20.97 to 36.39	0.550
<500 cells	-1.72	-70.67 to 67.23	0.960	12.40	-17.58 to -42.38	0.407
**HIV load**^d^						
>30 000	Reference			Reference		
5000–30 000	-49.83	-107.64 to 7.98	0.089	18.72	-10.06 to 47.50	0.195
< 5000	2.42	-87.47 to 92.31	0.957	36.85	-4.70 to 78.40	0.080
**Age** (years)	-0.27	-2.20 to 1.66	0.782	-1.23	-2.06 to -0.40	0.005
**Sex** (male versus female)	56.25	5.07 to 107.42	0.032	23.67	-0.87 to 48.21	0.058
**ART** (yes versus no)	-48.43	-100.41 to 3.56	0.067	-0.11	-23.87 to 23.64	0.992

^a^ Logarithm of copy number of parasites/ml blood.

^b, c^ cells/ml of blood,

^d^ Copy number of HIV virus/ml blood.

ART: Antiretroviral treatment.

A linear relationship was observed between antigenuria and parasitemia levels in both the unadjusted and adjusted regression model (Table 3). Interestingly, when levels of parasitemia were less than two logarithms, the expected increase in levels of antigenuria was 31.52 pg (95% CI: 17.04 to 46.01) per each increase in one logarithm of parasitemia (p<0.001, adjusted r^2^ = 0.84) (Table 3). Similarly, when levels of parasitemia were higher than two logarithms, the expected increase in antigenuria levels was 106.00 pg (95% CI: 85.13 to 126.85) per each increase in one logarithm of parasitemia (p<0.001, adjusted r^2^ = 0.84) (Table 3).

### Chunap, host factors and HIV infection

**Fig 3:**
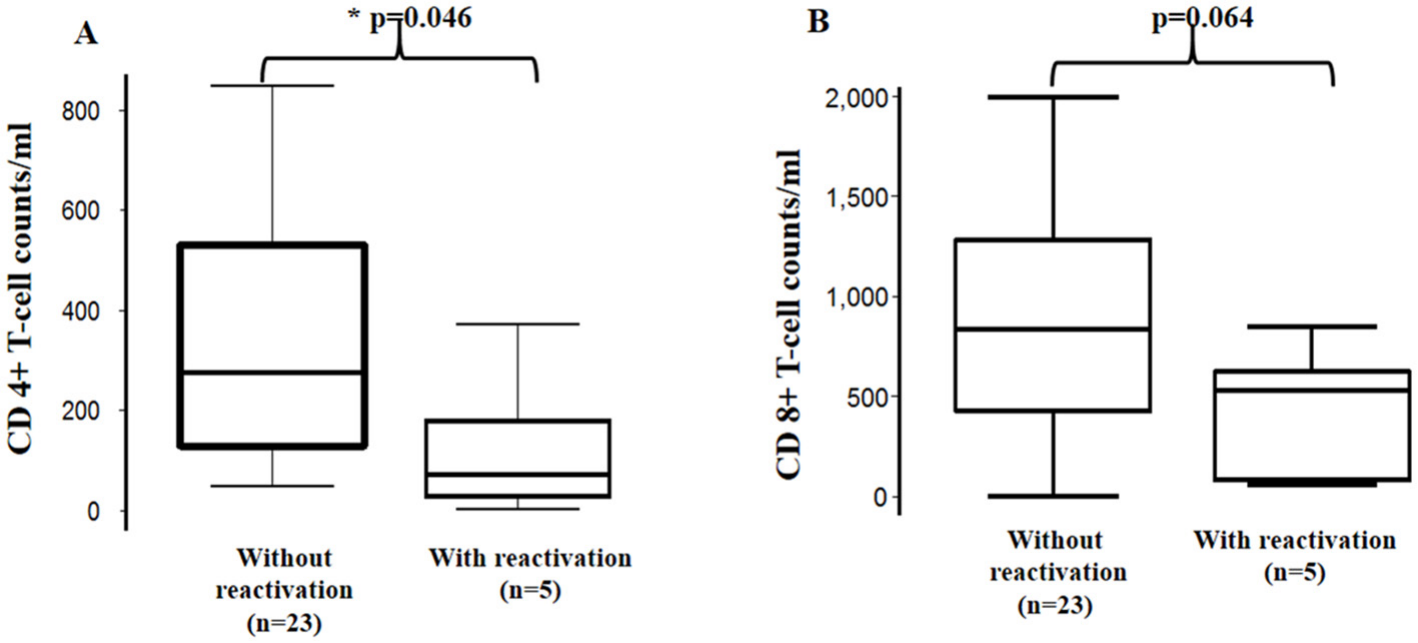
Counts of T-cell CD4+ and T-cell CD8+ in *T*. *cruzi* /HIV co-infected patients. (A) T-cell CD4+ and (B) T-cell CD8+ cells. Bottom and top limits of the boxes correspond to first and third quartiles, and the line inside the boxes represents the second quartile (median). P-values were calculated using T-test with unequal variances. * Statistically significant difference.

Among patients with Chagas disease, mean levels of CD4+ T-cell counts were significantly lower in patients with reactivation (131 cells, 95% CI: -56.54–318.54) compared to patients without reactivation (322.46 cells, 95% CI: 229.45–415.47) (t-test with unequal variances: p = 0.046) (Fig 3A). Clinical manifestations, parasitemia levels and immunosuppression status of each patient are shown in Table 4. Among patients with reactivation of Chagas disease, 3 showed clinical manifestations related with Chagas neurological disease, 1 patient had an intestinal perforation, and 3 patients did not show any clinical manifestations. An increase in levels of antigenuria of 36.08 pg (95% CI: 7.28 to 64.88, p = 0.016) in patients with < 200 CD4+ T-cell counts was observed in the adjusted linear regression model as compared to patients with >500 CD4+ T-cell counts (Table 3), but no differences were observed in the unadjusted model. No statistical associations were observed between antigenuria levels and CD+8 cells counts, HIV load, and ART in the regression model. There was a trend to lower mean levels of CD8+ T-cell counts between the two groups (reactivation group = 429.6 cells, Non-reactivation groups = 838.42 cells, t-test with unequal variances: p = 0.064) (Fig 3B). HIV viral loads were not statistically significantly different between patients with reactivation (mean: 134,303 copies/ml, 95% CI: -35,516 to 304,122) and without reactivation (mean: 121,761 copies/ml, 95% CI: -62,834 to 306,355) (Student’s t-test with unequal variances: p = 0.901).

**Table 4:** Clinical characteristics, antigenuria and parasitemia levels and immunosuppression status of HIV/*T*. *cruzi* co-infected patients.

**Reactivation cases: detectable parasitemia by microscopy**								
CODE	T-cell CD4+^a^	T-cell CD8+^b^	HIV load^c^	Chunap^d^	Parasite load^e^	Clinical manifestations^f^	ART^g^	Died
1	179	624	83653	432.1	5.6	Transverse myelitis	No	Yes
2	ND	ND	ND	396.7	5.4	CNS mass lesions	No	Yes
3	3	78	237684	255	3.5	None	Yes	No
4	ND	ND	ND	108.5	3.4	None	No	No
5	373	533	15301	142.9	2.9	None	No	No
6	71	852	17088	172.3	1.1	Meningitis	No	Yes
7	29	61	317789	188	0.9	Intestinal perforation	Yes	Yes
Mean	131	429.6	134303	242.2	3.3			
SD	151	348.7	136767.1	126.3	1.9			
**Moderate parasitemia: undetectable parasitemia by microscopy but qPCR positives**								
CODE	T-cell CD4+^a^	T-cell CD8+^b^	HIV load^c^	Chunap^d^	Parasite load^e^	Clinical manifestations^f^	ART^g^	Died
8	231	1033	120282	89.9	3.2	None	No	No
9	48	426	652536	22.7	2.2	None	No	No
10	497	ND	ND	54.3	2.1	None	Yes	No
11	ND	ND	ND	52.4	1.9	None	No	No
12	132	1615	69402	58.3	1.9	None	Yes	No
13	659	1527	ND	47.2	1.5	None	No	No
14	303	1093	7711	19.6	1.4	None	No	No
15	265	1336	68	42.7	1.4	None	Yes	No
16	328	898	40	99.9	1.3	None	Yes	No
17	532	1052	2706	24.9	1.2	None	No	No
18	168	417	121339	51.3	0.9	None	No	No
19	383	1052	ND	0	0.9	None	Yes	No
Mean	322.4	1044.9	121760.5	46.9	1.7			
SD	184.1	400.5	220801.3	32.2	0.6			
**Negative parasitemia: undetectable parasitemia by microscopy and qPCR**								
CODE	T-cell CD4+^a^	T-cell CD8+^b^	HIV load^c^	Chunap^d^	Parasite load^e^	Clinical manifestations^f^	ART^g^	Died
20	125	224	169815	22.3	0	Alterations in EKG	Yes	No
21	529	778	40	18.4	0	None	Yes	No
22	848	2000	3235	0	0	None	No	No
23	99	1352	1	0	0	None	Yes	No
24	562	ND	ND	0	0	None	Yes	No
25	68	1235	53	27.8	0	Alterations in EKG	Yes	No
26	563	ND	1576	0	0	None	Yes	No
27	105	491	3001633	0	0	None	Yes	No
28	210	562	39227	0	0	None	Yes	No
29	287	1579	ND	0	0	None	Yes	No
30	143	589	150	21.5	0	None	Yes	No
31	108	425	ND	32.3	0	None	Yes	Yes
Mean	303.9	923.5	357303.3	24.5				
SD	256.4	582.9	993176.6	5.5				

^a, b^ cells/ml of blood.

^c^ copy number of HIV virus/ml blood.

^d^ pg of *T*.*cruzi* antigens.

^e^ Copy number of parasites/ml blood.

^f^ Clinical manifestations related to Chagas disease.

^g^ Antiretroviral treatment.

## Discussion

Reactivation of Chagas disease in HIV patients is a serious medical condition, which is often life-threatening. Early diagnosis of Chagas reactivation and prompt treatment can be life-saving. However, current methods are based on microscopy that have sub-optimal sensitivity that do not detect reactivation until parasite loads are very high. A sensitive, non-invasive test is needed to monitor increase in parasitemia levels and predict risk of reactivation. In this study we introduce the potential use of a nanoparticle-based tool to monitor *T*. *cruzi* infection in urine of HIV patients.

### Chagas and HIV infection

No differences were observed in HIV loads, CD4+ cell and CD8+ cell count, and presence of other co-infections between HIV patients with and without Chagas disease. Among patients with positive serology for Chagas disease, patients with reactivation of Chagas disease had low levels of CD4+ T-cells counts compared to patients without reactivation, as previously observed [15]. The percentage of mortality attributed to Chagas disease was 16.12% (5/31), which is similar to the reported in longitudinal studies (15.09%, 8/53) [10].

Clinical manifestations related to Chagas disease were observed in the nervous system [7, 10]. In the case of the patient with intestinal perforation we were not able to perform a biopsy analysis to make the confirmatory diagnosis; however, intestinal perforation is a complication described in patients with digestive system abnormalities associated with Chagas disease [36]. The presence of asymptomatic reactivation has been previously described and is thought to be an early stage of Chagas reactivation and a risk factor of HIV progression [10, 37].

### Performance of Chunap in the diagnosis of Chagas disease

An IgM monoclonal antibody against *T*. *cruzi* lipophosphoglycan (LPG) was used in this study for antigen detection. The *T*. *cruzi* LPG is a surface glycoconjugate which is composed mainly of glucosamine, sialic acid and galactosamine in the carbohydrate portion, and of alkylacylphosphatidylinositol in the lipid portion [38]. Although this antigen has been primarily characterized in the epimastigote form, it could be an important component of the trypomastigote form, and could be used during cell recognition, invasion, and immune suppression of the host [38]. This antibody recognizes two bands of 42 kDa and 82 kDa in the trypomastigote secretory-excretory antigen and crude sonicated trypomastigotes [26].

The percentage of antigenuria positives detected by Chunap (74%) among those with positive serology is similar to that reported before in chronic infected patients (60%-84%) [39–40]. Two false positive results were found in urine samples of patients with negative serology; one of whom was also co-infected with *M*. *tuberculosis*. We hypothesize that the presence of false positives could be explained by the existence of other co-infections. In the case of tuberculosis, the mycobacterial lipoarabinomannan has been detected in urine samples of HIV co-infected patients [41]. The lipophosphoglycan is also found in other infectious agents such as *Leishmania*, *Trichmonas*, and *Pneumocystis*, but the anti-IgM LPG antibody used in this study does not show cross-reaction with the LPG from *Leishmania* and *Trichomonas* species [38]. However, further studies are needed in order to assess cross-reaction with other parasites that are commonly present in HIV, such as *Toxoplasma gondii*, and bacteria and fungi such as *M*. *tuberculosis* and *Cryptococcus neoformans*.

### Chunap and relationship with parasitemia levels

Definition of a parasite load threshold for prediction of reactivation could be used as a guide in the use of anti-trypanosomal therapy. The early increase in parasitemia may not be symptomatic as previously described in one prospective study [10], so monitoring for asymptomatic parasitemia may permit early detection of reactivation. This could lead to accelerating the initiation of anti-trypanosomal therapy, which could prevent irreversible damage or death.

Levels of parasitemia, determined by qPCR, were higher in patients that had reactivated Chagas disease than in those without reactivation. However, there was a high variability of parasitemia levels between individuals. This observation was also reported by a previous study (mean ± SD in reactivation cases VS non-reactivation: 12,584.96 ± 11,368.35 VS 10.43 ± 3.53, respectively) [15]. The high variability in parasitemia levels detected by qPCR could be explained by differences in the strains of *T*. *cruzi*, and by the inability to distinguish DNA from living and dying parasites [15]. Although there was a wide SD found among antigenuria levels, the variability was less than that seen in parasitemia. This could be explained due to lipophosphoglycan only being excreted by living parasites [38].

In this study, reactivation was defined as the detection of circulating parasites in blood by microscopy with or without the presence of clinical manifestations [10]. This definition has limitations because of the lack of sensitivity and reproducibility of microscopy. As observed by us and others, patients with positive microscopy could have low levels of parasitemia by qPCR (and vice-versa), suggesting a poor correlation between microscopy and qPCR. For example, in the case of patient number 9 in Table 4, the levels of parasitemia and viremia were high, and the CD4+ counts was low. Yet this patient had undetectable parasitemia by microscopy, thus not meeting the reactivation criteria. A better definition of Chagas reactivation is needed so that immunosuppression status and HIV load are also taken into consideration. This will guide clinicians in case management.

In the guinea pig model of Chagas disease and congenital Chagas disease, the presence of antigens in urine is correlated with high levels of parasitemia [19, 26]. In this study we demonstrate that antigenuria levels are positively related with parasitemia levels in humans. Interestingly, the increase in levels of antigenuria was greater among patients with higher levels of parasitemia (> 2 logarithm copy number parasites/ml). All patients with levels of antigenuria higher than 105 pg were patients that showed reactivation. In this cross-sectional study, we have evaluated patients that have reactivated Chagas disease, a longitudinal study will be necessary to determine a cut-off of antigenuria that predicts reactivation and could be used in low-resource settings where serial evaluations of samples are logistically difficult [15].

### Chunap and host factors

Antigenuria levels were significantly higher in patients with < 200 CD4+ T-cells, but only in the adjusted model, suggesting possible confounding effects of HIV loads, treatment, age, and sex. In contrast to a previous study, we could not find statistically significant differences between levels of parasitemia or antigenuria, and HIV load and CD8+ T-cells levels [15]. Limitations of this study include possible confounding effects of HIV treatment and the small sample size. Although we adjusted by the use of ART to make statistical comparisons, we could not account for the duration of ART. ART drugs have a direct and more immediate effect on HIV loads as compared to levels of CD4+ T-cells and CD8+ T-cells [15].

Among patients with Chagas disease, young age was found to be a risk factor for high antigenuria levels in the adjusted model. However, this association could be influenced by the time of diagnosis of HIV infection and ART.

The Chunap reached sensitivity levels comparable to the more expensive and time consuming standard of care micromethod and qPCR. The feasibility data presented herein provide evidence that Chunap is a promising tool to improve current Chagas diagnostic algorithm in clinical settings. Furthermore, in a previous study we demonstrated that Chunap protect *T*. *cruzi* antigens from degradation [26].

In future studies, poly(NIPAm/TB) particles will be magnetized in order to simplify the clinical diagnostic process and extend accessibility. Sensitivity can be further improved by increasing the volume of urine analyzed. In conclusion, an antigen urine test for monitoring Chagas reactivation in HIV patients would be highly desirable for these reasons: a) levels of antigens in urine are related to levels of parasitemia b) antigenuria levels are less variable compared to levels of parasitemia c) urine is a preferred, less-infectious, non-invasively collected biological fluid that is more acceptable by patients (especially if continuous samples are needed), and d) antigen testing can be scaled to a rapid, point of care test that can be performed in low-equipped laboratories.

### Members of the Chagas/HIV Working Group in Bolivia and Peru

Jean Cabeza, Roni Colanzi, Daniel Lozano, Gonzalo Borda, Gerson Galdos, Lisbeth Ferrufino, Louisa Messenger, Rosmery Gross, Leny Sanchez, Omar Gandarilla, Maurus Dorn, and Helena Jahuira.

## Supporting Information

S1 Fig Linear regression and 95% CI for densitometric quantification of trypomastigote excretory-secretory antigen (TESA) spiked in normal human urine (concentrations 5 pg, 10 pg, 50 pg, 250 pg, 500 pg) and concentrated and detected using Chunap. (TIF)

S1 Checklist STARD checklist. Use of Chunap for detection of Chagas disease in *T*.*cruzi*/HIV co-infected patients.
(PDF)

S1 Diagram STARD diagram. Use of Chunap for detection of Chagas disease in *T*.*cruzi*/HIV co-infected patients. * Confirmation of *T*. *cruzi* infection was based on positive results by 2 or more serological tests for detection of anti- *T*. *cruzi* IgG antibodies. ** Reactivation of Chagas disease was based on the detection of circulating parasites by microscopy.
(TIF)
