# Supplementary figures and images for: Use of a Chagas Urine Nanoparticle Test (Chunap) to Correlate with Parasitemia Levels in T. cruzi/HIV Co-infected Patients
Source: PLoS Negl Trop Dis. 2016 Feb 26;10(2):e0004407. doi: 10.1371/journal.pntd.0004407 (PMC4768913; doi:10.1371/journal.pntd.0004407)

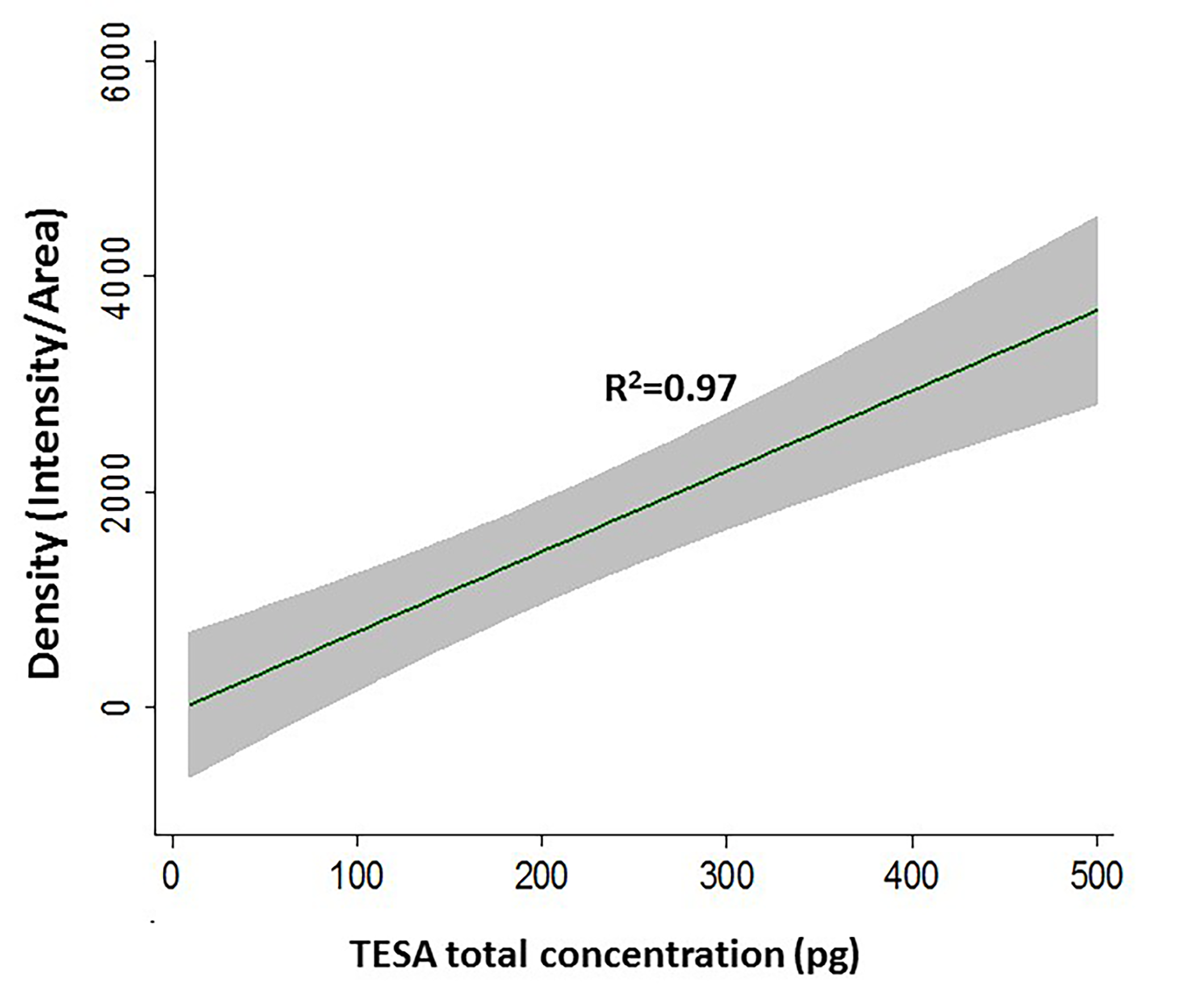

Supplement: S1 Fig — (TIF) [file pntd.0004407.s001.tif]

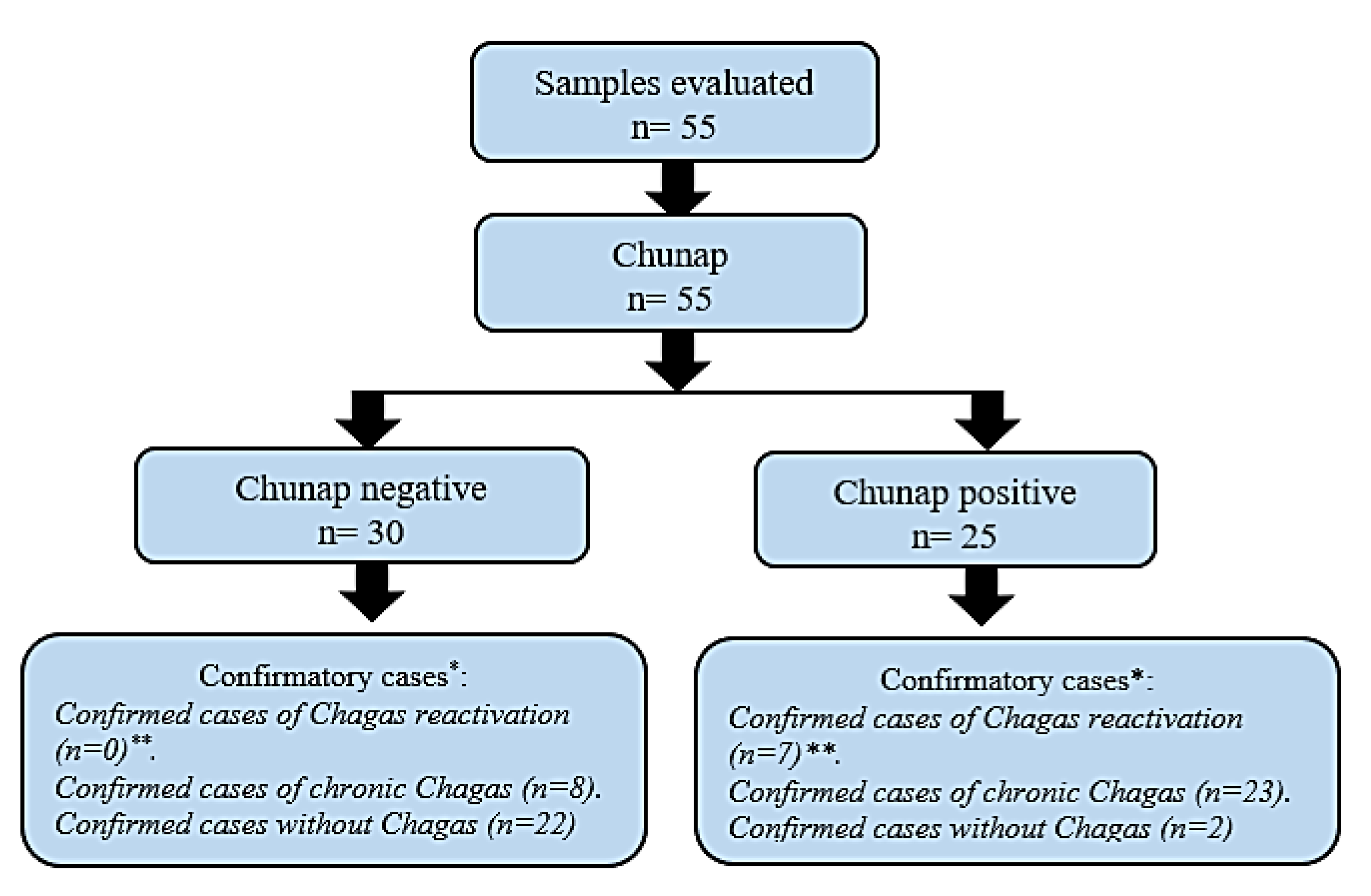

Supplement: S1 Diagram — Use of Chunap for detection of Chagas disease in T.cruzi/HIV co-infected patients. * Confirmation of T. cruzi infection was based on positive results by 2 or more serological tests for detection of anti- T. cruzi IgG antibodies. ** Reactivation of Chagas disease was based on the detection of circulating parasites by microscopy. (TIF) [file pntd.0004407.s003.tif]
